# Wiping out MRSA: effect of introducing a universal disinfection wipe in a large UK teaching hospital

**DOI:** 10.1186/s13756-018-0445-7

**Published:** 2018-12-19

**Authors:** Mark I. Garvey, Martyn A. C. Wilkinson, Craig W. Bradley, Kerry L. Holden, Elisabeth Holden

**Affiliations:** 1 University Hospitals Birmingham NHS Foundation Trust, Queen Elizabeth Hospital Birmingham, Edgbaston, Birmingham, B15 2WB England; 2 Institute of Microbiology and Infection, The University of Birmingham, Edgbaston, Birmingham, England; 3 Gloucestershire Hospital’s NHS Foundation Trust, Gloucester Royal Hospital, Great Western Road, Gloucester, GL1 3NN England

**Keywords:** Meticillin-resistant *Staphylococcus aureus*, MRSA bacteraemias, MRSA acquisitions, Disinfection wipes

## Abstract

**Background:**

Contamination of the inanimate environment around patients constitutes an important reservoir of MRSA. Here we describe the effect of introducing a universal disinfection wipe in all wards on the rates of MRSA acquisitions and bacteraemias across a large UK teaching hospital.

**Methods:**

A segmented Poisson regression model was used to detect any significant changes in the monthly numbers per 100,000 bed days of MRSA acquisitions and bacteraemias from April 2013 - December 2017 across QEHB.

**Results:**

From April 2013 to April 2016, cleaning of ward areas and multi-use patient equipment by nursing staff consisted of a two-wipe system. Firstly, a detergent wipe was used, which was followed by a disinfection step using an alcohol wipe. In May 2016, QEHB discontinued the use of a two-wipe system for cleaning and changed to a one wipe system utilising a combined cleaning and disinfection wipe containing a quaternary ammonium compound. The segmented Poisson regression model demonstrated that the rate of MRSA acquisition/100,000 patient bed days was affected by the introduction of the new wiping regime (20.7 to 9.4 per 100,000 patient bed days; p <0.005).

**Discussion:**

Using a Poisson model we demonstrated that the average hospital acquisition rate of MRSA/100,000 patient bed days reduced by 6.3% per month after the introduction of the new universal wipe.

**Conclusion:**

We suggest that using a simple one wipe system for nurse cleaning is an effective strategy to reduce the spread and incidence of healthcare associated MRSA.

## Introduction

*Staphylococcus aureus* is a major cause of healthcare-associated infection worldwide [1–3]. Meticillin-resistant *S. aureus* (MRSA) has become prevalent in most parts of the world [1–4]. Despite its decline in incidence in several European countries, MRSA infection remains a major cause of avoidable morbidity and mortality in patients admitted to hospital [1–4]. It results in increased length of hospital stay, risk of death and treatment costs [4], with colonized and infected patients acting as reservoirs for the spread of MRSA within hospitals [5, 6]. Isolation and decolonization are the two main targeted control measures for reducing transmission of MRSA within hospitals [7, 8].

It is acknowledged that the healthcare environment plays a key role in facilitating the transmission of important pathogens responsible for healthcare-associated infections [9]. These pathogens include vancomycin-resistant enterococci (VRE), *Clostridium difficile* and MRSA [9]. Such organisms are able to survive in the environment for many days, posing an ongoing risk of transmission and acquisition by hospital patients [10]. In recent years, there has been more interest from infection control staff, clinicians, health planners and government on maintaining a clean environment [9–11]. Contamination of the inanimate environment around patients constitutes an important reservoir of multi drug resistant organisms, with the risk of healthcare-associated infection increasing significantly if the patient previously occupying the room had MRSA, VRE, *C. difficile* or other multidrug resistant pathogen [11]. Patients in rooms previously occupied by MRSA colonized or infected patients had a twofold increased risk of MRSA acquisition [11, 12]. Garvey *et al.,* (2017) found that during a PVL positive MRSA outbreak, up to 40% of the environmental areas on a ward sampled contained MRSA [13]. Even with enhanced cleaning, the PVL positive MRSA strain was not eliminated from the environment [13]. It is not uncommon for hand hygiene and the environment to play a role in the transmission of MRSA in a hospital setting; various reports have previously detailed these transmission routes [12, 14].

Between April 2013 to April 2016, ward areas at Queen Elizabeth Hospitals Birmingham (QEHB) part of University hospitals Birmingham were cleaned by nursing staff using a two-wipe system. Specifically, this included routine cleans of the ward environment, patient equipment and post discharge cleaning of bed spaces and beds of patients without known nosocomial pathogens. This entailed using, firstly a detergent wipe, followed by a disinfection step using an alcohol wipe. From May 2016, QEHB discontinued the two-wipe system, changing to a one-wipe system utilising a combined cleaning and disinfection wipe. We report the effect of introducing a universal disinfection wipe in all wards on the rates of MRSA acquisition across QEHB.

## Materials and methods

### Setting

Queen Elizabeth Hospital Birmingham (QEHB), part of University Hospitals Birmingham (UHB) NHS Foundation Trust is a tertiary referral teaching hospital in Birmingham, UK that provides clinical services to nearly one million patients every year [8].

### MRSA screening

MRSA screening of all emergency, elective surgical patients and day case patients (deemed to have high risk procedures such as surgery) admitted to UHB is standard practice. Only day case patients whom receive minor procedures such as endosocpy are not screened. Swabs are taken from the nose, groin and throat as well as any wounds or sites of invasive devices. Inpatients with greater than 28 days’ stay are rescreened every 4 weeks. Only one change to MRSA screening has been undertaken at QEHB during this report, in August 2014 MRSA screening to low risk patients such as day-case patients was stopped.

### MRSA acquisitions

At QEHB a patient is defined as acquiring MRSA if they have a negative admission screen and then have MRSA isolated from a subsequent screen or clinical specimen, 48 hours after admission. Only MRSA acquisitions at QEHB were included in the analysis. All MRSA acquisitions are identified as previously described by Garvey *et al.,* (2016) [14, 15].

### MRSA bacteraemia

All MRSA bacteraemias were attributed as healthcare associated or non-healthcare associated using the criteria of the Centres for Disease Control and Prevention/National Healthcare Safety Network for national reporting purposes [16]. All bacteraemias then go through a Post Infection Review process for assignment of apportionment either to the acute Hospital, Clinical Commissioning Group (CCG, community based) or ‘third party’, regardless of attribution to healthcare or non-healthcare associated [16]. The third part of the amendment allows for the assignment of a case of MRSA bloodstream infection to a ‘third party’ through the arbitration process lead by the Regional Director of Nursing or Regional Medical Director. Third party assignment provides an acknowledgement of the complex nature of MRSA bloodstream infections being reported which previously may have been allocated by default to acute hospitals or CCGs who were not involved in the patients care or who can provide a strong case following the Post Infection Review that there were no possible failings in patient care. Only bacteraemias that were assigned to the acute hospital (QEHB) were included in the analysis [16]. Similar to acquisitions, all MRSA bacteraemias are molecularly typed to help identify transmission links [14, 15].

### Current cleaning programme

The hospital utilises an environmental cleaning procedure consisting of four different grades. Red and amber cleans are employed when the prior occupant of the room or bed space was known or suspected of having an alert organism or diarrhoea. An alert organism is classified as a transmissible nosocomial pathogen including, but not limited to: *C. difficile*, norovirus, MRSA, extended spectrum β-lactamase (ESBL) producing organisms, VRE, Influenza, *Mycobacterium tuberculosis* and *Streptococcus pyogenes*. The cleaning methods for these grades require additional training for the operators; for example, housekeeping assistants receive training for curtain changes, use of a hydrogen peroxide misting system, cleaning of sinks and making up the chemicals. Included in both red and amber cleans, are a surface and equipment clean using a chlorine- and detergent-based agent (ChlorClean®, Guest Medical Limited, Edenbridge, UK) with disposable cloths, and the curtains are changed. In addition, the red clean involves the application of 6% w/v hydrogen peroxide misting by aerosolisation (Oxyfarm, Glasgow, UK). For cleans where the patient was not known or suspected of harbouring an alert organism, a green clean is carried out using a single detergent/disinfectant wipe (wipe A – Table 1). Nursing staff are responsible for green cleans, which include a surface and equipment clean using a single detergent/disinfectant wipe (wipe A - Table 1). All bed-side equipment are wiped until visibly-clean, as well as the bed frame, touch points including the nurse-call handset, door handles and bed-side chair. The curtains are inspected for signs of soiling and are changed if required. Nurses have been trained in how to store and use the universal wipes and regularly use a new wipe when, either the existing wipe is dry, or moving on to a new piece of equipment.

**Table 1:** Wipes used at QEHB between April 13 to December 17 including chemical composition of wipes

Wipe	Detergent/ disinfectant wipe	Contents
A	Detergent/ disinfectant wipe (Clinell Universal Sanitising Wipes, GAMA Healthcare Limited, UK)	Bezalkonium chloride ≤0.5%, Didecyl dimethyl ammonium chloride ≤0.5%, Polyhexamethylene biguanide (PHMB) ≤0.10%, Water >75%, Additives each <1%
B	Detergent wipe	Phenoxyethanol <1%, Alkyl polyglycoside <0.2%, Diethylene glycol <0.1%, 2-Octyl-2H-Isothiazol-3-one <0.01%
C	Alcohol wipe	Propan-2-ol 50-80%

### Cleaning programme pre-May 2016

Compared to the current protocol, the only difference in cleaning involved the green clean, where two wipes were used instead of one. From April 2013 to April 2016, the cleaning of ward areas by nursing staff involved firstly, a detergent wipe, followed by a disinfection step using an alcohol wipe (wipes B and C, respectively - Table 1). Post May 2016 QEHB moved away from a two-wipe system to a universal one wipe system. The decision to choose wipe A was based on the ease of us, log reduction killing against a range of nosocomial microorganisms and staff preference (data not shown). Before implementation and hospital wide use of wipe A, several wipes were tested for efficacy in cleaning against a range of nosocomial organisms with the most efficacious (top two wipes) at reducing the number of nosocomial microorganisms being trialled on two wards for staff feedback. The wipe most preferred by staff based on a questionnaire was then chosen for use across QEHB.

### Education

Before the implementation of the new single disinfectant wipe (wipe A), an education package spanning one month was implemented throughout QEHB. The training package was delivered by trained Infection Control Nurses from GAMA healthcare (GAMA healthcare, UK) and involved teaching ward nurses the following: how to use the universal wipe for a surface and equipment clean; equipment to be cleaned, including all bed-side equipment, the bed frame, touch points including the nurse-call handset, door handles and bed-side chair. Nurses were also trained how to store and use the universal wipes, and to use a new wipe when either the old wipe became dry or moving on to a new piece of equipment. This practice was audited by Infection Prevention and Control Nurses. Between April 2016 – December 2017 1160 frontline healthcare workers primarily nurses were trained in how to use the new wipe.

### Audits

Regular audits were undertaken including monitoring hand hygiene compliance, monitoring the appropriate use of personal protective equipment and monitoring environmental cleanliness. Hand hygiene audits are undertaken by the wards either daily or weekly and validated by the Infection prevention and Control Team. PPE audits are undertaken monthly by the Infection prevention and control team. Environmental cleanliness audits are undertaken monthly by the facilities/estates team and validated by the Infection prevention and control Team.

### ICU decolonization timeline

As described previously by Bradley *et al.,* (2017) before August 2014 all ICU patients at UHB received universal MRSA decolonization therapy regardless of their MRSA status [8]. Between August 2014-December 2015 universal decolonization therapy of all ICU patients was withdrawn and only MRSA positive patients received MRSA decolonization therapy [8]. In December 2015 UHB reintroduced universal MRSA decolonization therapy to all ICU patients regardless of their MRSA status [8].

### Statistical analyses

Segmented Poisson regression models containing offsets for patient bed days were used to detect any significant changes in the rate of MRSA acquisitions and bacteraemias from April 2013 – December 2017. There were no other changes to the Infection Prevention and Control policy or practice during this period. ‘Full’ models were constructed containing terms for changes in level and gradient associated with the following potentially-important interventions: the withdrawal and reintroduction of universal decolonization of patients in the intensive care unit and the change in wiping regime. Backward step-wise regression was performed on the ‘full’ Poisson models, with the Akaike Information Criterion used to select the models that best fit the data, whilst guarding against over-fitting. All statistical analyses were performed using R version 3.4.3 [17, 18].

## Results

### MRSA acquisitions

**Fig. 1:**
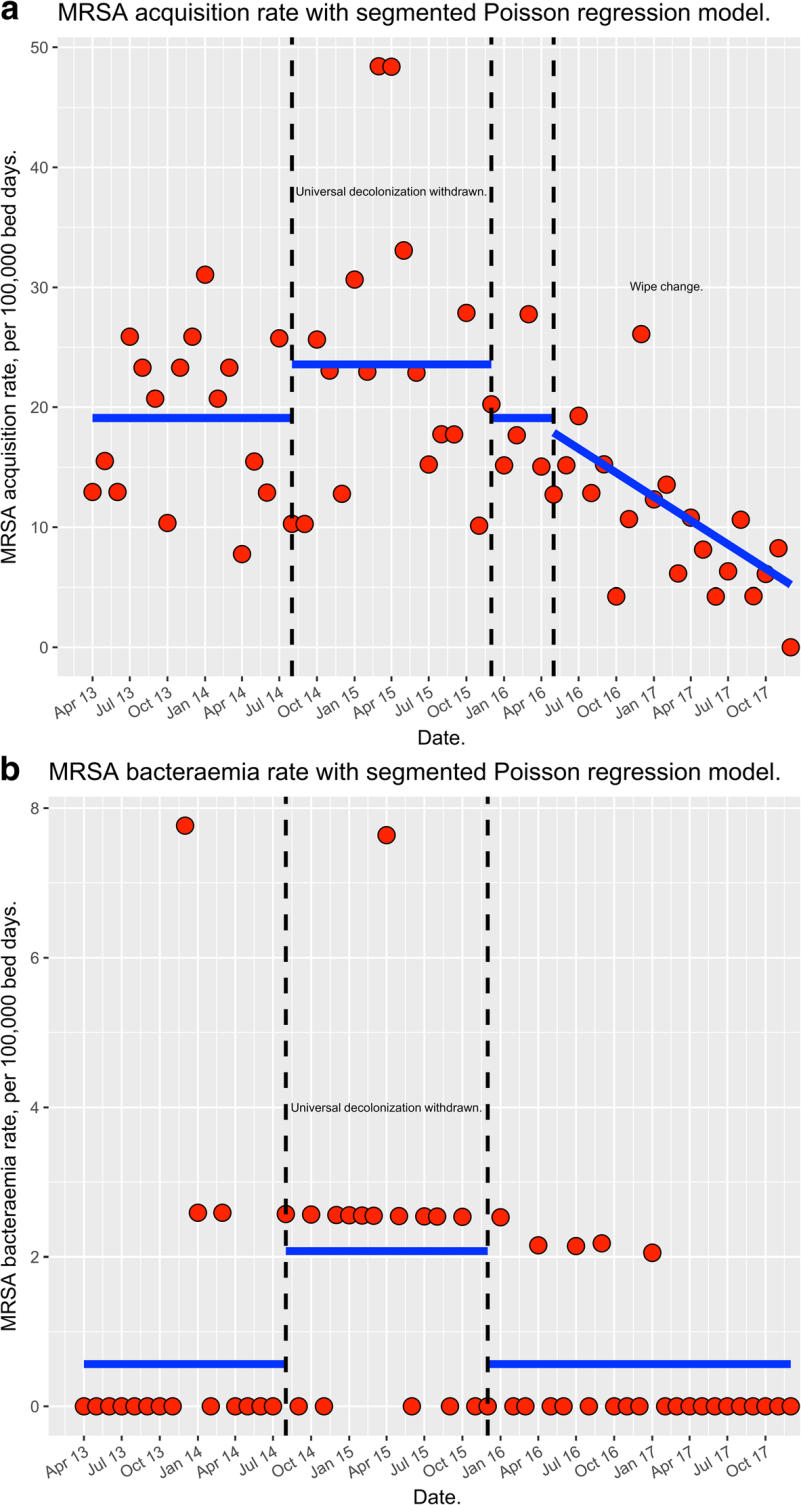
Using a segmented Poisson regression model changes in hospital wide monthly MRSA acquisition rates (**a**) and MRSA bacteraemia rates (**b**) per 100,000 bed days between April 2013-December 2017. Key: A two wipe regime for nurse led cleans was undertaken at QEHB between April 2013 to April 2016; a one wipe regime for nurse led cleans was undertaken at QEHB between May 2016 to December 2017; the removal of universal MRSA decolonization therapy in the ICU at QEHB occurred during August 2014-December 2016; the reintroduction of universal MRSA decolonization therapy in the ICU at QEHB occurred between January-December 2017. The dotted lines represent the infection prevention and control interventions. The blue lines represent the mean values predicted by the Poisson regression model

We analysed QEHBs acquisition rate of MRSA per 100,000 patient bed days using a segmented Poisson regression model. The number of MRSA acquisitions across QEHB between April 2013 and April 2016 averaged at around 20.7 per 100,000 patient bed days (Table 2). Following the introduction of a one wipe system the number of MRSA acquisitions across QEHB decreased from 20.7 to 9.4 per 100,000 patient bed days (Table 2 and Figure 1a; 310 vs 93 acquisitions). The optimum Poisson model demonstrates that the rate of acquisition of MRSA per 100,000 patient bed days was affected by the withdrawal of universal decolonization (p = 0.057) and by the introduction of a new one wipe regime (p = 2.89 x 10^-8^). The evidence for the effect of the new wiping regime was much stronger. The model demonstrates that the average trust acquisition rate of MRSA per 100,000 patient bed days reduced by 6.3% per month after the introduction of the new wipes (Fig. 1a).

**Table 2:** Total number of patients admitted to ICU and to the hospital during the 3 study periods. The total number of MRSA acquisitions and bacteraemias across QEHB are shown during the 3 study periods

	April 13 - August 14	August 14 - March 16	April 16 - December 17
Bed days - Critical Care	35,595	40,165	51126
Bed days - QEHB Total	550,107	606,820	989,724
MRSA acquisitions	119	191	93
MRSA bacteraemias	5	16	2

### MRSA bacteraemia

The MRSA bacteraemia rate per 100,000 patient bed days for the same period was also analysed using a segmented Poisson regression model. The model demonstrated that there was a 270% increase in the average MRSA bacteraemia rate per 100,000 patient bed days during the period of cessation of universal decolonization (p = 0.00203; Fig. 1b). The model demonstrated no change in the rate of MRSA bacteraemias associated with introducing a new one wipe regime.

### Auditing

Audits revealed no significant changes in hand hygiene compliance; appropriate use of personal protective equipment (PPE) or environmental cleanliness during the study (data not shown). Any failures in practice education sessions are undertaken to address any issues. MRSA screening for emergency and elective patients averages at 90% and 92% respectively, over the past five years at QEHB. At QEHB there is electronic prescribing so all patients who are MRSA positive receive decolonization therapy, thereby no prescribing patterns were altered during this study.

## Discussion

The English Department of Health (DoH) introduced universal mandatory MRSA screening of all elective and emergency admissions to English hospitals in 2010 [19]. In 2012 the DoH commissioned a national audit (National One Week Prevalence Audit of MRSA) to review implementation and impact of patient management from universal MRSA screening [20]. The audit found implementation of universal screening was poor, MRSA admission prevalence (new cases) was low and suggested UK hospitals should seek to improve implementation of current MRSA screening policy [20]. The report made recommendations to stop routine screening in large teaching Hospitals [20]. In response to the National One Week Prevalence Audit of MRSA, QEHB implemented a revised strategy for the control of MRSA [20]. Before 2014 all ICU patients at QEHB received universal MRSA decolonization therapy regardless of their MRSA status [8]. In August 2014 QEHB discontinued the use of universal decolonization in the ICU and stopped MRSA screening to low-risk patients such as day cases having minor procedures such as endoscopy [8]. A breakpoint model identified an increase in MRSA bacteraemias and acquisitions across QEHB, subsequent to the withdrawal of universal decolonization [8]. As a result of increased rates of MRSA, universal decolonization was reintroduced into the ICU resulting in a reduction of MRSA bacteriaemias and acquisitions across QEHB [8]. Here we follow on from this work and detail the effect of introducing a universal detergent/disinfection wipe for nurse-led cleans in all wards on the rates of MRSA acquisitions across QEHB.

A segmented Poisson regression model suggests that the rate of acquisition of MRSA per 100,000 patient bed days was affected by the withdrawal of universal decolonization, as per the work by Bradley *et al.,* (2017), and unexpectantly by the introduction of a new wiping regime [8]. Whilst the evidence for the effect of universal decolonization on the number of MRSA acquisitions was statistically rather weak, the optimum model suggested that the average rate of acquisition of MRSA per 100,000 patient bed days increased by 23% during the period of withdrawal of MRSA decolonization on ICU. The evidence for the effect of the new universal wipe regime was much stronger. The data suggest the use of a one wipe regime is associated with reducing the incidence of healthcare associated MRSA. It is not surprising that cleaning and disinfection could be so important in the reduction of MRSA acquisitions across QEHB. MRSA has been demonstrated to exist in the healthcare environment and cleaning has been shown to reduce the levels of MRSA in the environment [12, 13]. A systematic review by Dancer *et al.,* (2012) revealed that MRSA is found in the healthcare environment and reductions in the environmental burden have been shown through detergent and disinfectant regimes [9]. The use of disinfectant wipes is common practice within UK hospitals for the decontamination of environmental surfaces coming into contact with patients, for example common hand touch point areas, as well as patient equipment [21, 22]. This is often undertaken by nursing staff, with more specialised room cleans for infected patients undertaken by dedicated cleaning teams [21, 22]. This is true of the cleaning practice at QEHB, where cleans of rooms for non-infected patients are undertaken by nursing staff, predominantly using disinfectant wipes. Kundrapu *et al.,* (2012) demonstrated that the decontamination of environmental surfaces can reduce the risk of contamination of the hands of healthcare personnel [23]. A cleaner environment including patient equipment will result in lower levels of MRSA being transferred to healthcare workers’ hands and subsequently to patients. Further work is needed to elucidate whether the new wipe regime reduced the amount of environmental contamination with MRSA; environmental monitoring before and after implementation of the new wipe regime could have been one of the ways to observe this. The effect of the new wipe regime on the rate of MRSA acquisitions was an unexpected finding, further work is needed to identify if the implementation of a new wipe had an effect on the rate of patient acquisitions of other nosocomial alert organisms.

The optimum Poisson model provided clear evidence that the rate of MRSA bacteraemia per 100,000 patient bed days was affected by the withdrawal of universal decolonization as previously described by Bradley *et al.,* (2017) [8]. The number of MRSA bacteraemias was unaffected by the change in wipe regime. The authors hypothesise the patients most at risk of developing an MRSA bacteraemia are patients which pass through the ICU. The percentage of inpatients passing through the 100 bedded ICU at QEHB is 5-10% and account for 5.5% of the total bed days. By decolonising this group of patients, the authors believe this resulted in the reduction of MRSA bacteraemias independent of the change in wipe regime.

Detergent wipes are formulated to remove contamination from surfaces (i.e. to physically clean) [24–26]. Disinfectant wipes contain specific antimicrobial agent(s) to inactivate the bioburden on surfaces, which may contain infectious microorganisms and bodily fluids [24–26]. Due to the variety of detergent and disinfectant wipes available between April 2013 and April 2016, guidance by the Royal College of Nursing dictated that the manufacturer’s instructions were followed when using wipes [21, 22, 24–26]. This often entailed a detergent wipe to be used to clean the surface of gross debris/heavy soil, followed by the use of a disinfectant wipe to disinfect the surface [21, 22]. A number of studies have advocated a ‘1 wipe, 1 surface, 1 direction’ approach, which is considered to be applicable for use in practice [21, 22]. Rahm *et al.,* (2015) demonstrated that no detergent wipe removes all contamination and if used incorrectly, contamination can be transferred to subsequent surfaces [25]. Between April 2013 and April 2016, QEHB used a two-wipe system involving a detergent wipe (wipe B) and an alcohol disinfectant wipe (wipe C). In April 2016 QEHB changed to a one wipe detergent and disinfection wipe (wipe A) for ease of use. Following the introduction, the number of MRSA acquisitions across UHB decreased from 20.7 to 9.4 per 100,000 patient bed days. It is not surprising moving from a two-wipe system to a one wipe system would affect the cleanliness of the hospital environment. Borg (2014) described infection control as a behavioural science, and here we show a change in practice to something which is simpler can have a result [27]. Using a simpler system would be easy to flow for busy healthcare workers. Borg (2014) discusses the Hofstede model for behavioural change with the key to improved infection control and prevention behaviour through effective education, motivation and system change [27]. By moving to something simpler it is not unsurprising that a one wipe system would be taken up more readily by staff and undertaken appropriately. The use of combination wipes fits well with a human factors approach, being available at the point of use and maximising the opportunity for correct practice. Further work is needed to elucidate the behavioural science of healthcare workers into cleaning. Although behaviour will be a factor in the observed reduction in MRSA acquisitions seen across QEHB it should be noted that the wipe QEHB changed to might be more effective. Ramm *et al.,* (2015) demonstrated that different types of detergent wipes and different wipe substrates can be more effective at preventing the transfer of MRSA [25]. Changing to a more effective wipe could also explain the results seen in the present study. The final factor that could have contributed in a reduction of MRSA acquisitions across QEHB is training and education associated with the new wipe change. Borg (2014) discusses that education plays a role in infection prevention and control behaviour [27]. Training healthcare workers in how to use the new wipe would improve how areas within QEHB are cleaned. The numbers of nurses trained at QEHB in how to use wipe A was 1160 between April 2016- December 2017. In total there are around 2500 nurses employed within QEHB so not all staff have been trained in the use of the wipe. The effect of training is well documented in the literature however further work is needed to elucidate the true impact of cleaning training packages in the healthcare setting [28]. The effects of training are usually temporary however improvement trends continues after training packages have been implemented [28]. The effect of training could also in part explain the observations seen in the current report. It also must be noted that the authors did not look at the effect of trained vs non-trained nurses in the different specialities where differences in training could have been observed.

## Conclusion

Strengths of this study include the use of segmented Poisson regression models which allow for changes in gradient as well as level, and so can assess the cumulative impact of any intervention more clearly [17]. Importantly the study was performed in a low prevalence setting, with a baseline rate of 20.7 MRSA acquisitions per 100,000 patients. One cannot rule out the possibility that other factors other than changing to a new detergent/disinfectant wipe may partly explain the difference seen in the rates of MRSA acquisitions across QEHB. In consideration of other variables that could have contributed to the increase in MRSA, we examined whether poor infection control procedures had a part to play. We found that there was no evidence that hand hygiene; use of personal protective equipment and environmental cleanliness had seen reductions in compliance. Audit data provided from QEHB demonstrates comparable rates during periods of the change from a two-wipe nurse led cleaning regime to a one wipe regime. The authors did not undertake any analysis of the above factors which could have contributed to the observed result. Further statistical models are needed to identify if other confounders may explain the results seen in this report. This report suggests that there is an association between the use of a one wipe regime and the reduction in spread and incidence of healthcare associated MRSA.
